# Assessing Parental Understanding and Approaches of Preventive Dental Care for Children: A Cross-Sectional Study in Saudi Arabia

**DOI:** 10.7759/cureus.77273

**Published:** 2025-01-11

**Authors:** Nouf M Altamimi, Nashwa A Bushara

**Affiliations:** 1 College of Dentistry, University of Hail, Hail, SAU

**Keywords:** children’s oral health, cross-sectional survey, dental education, dental visits, oral hygiene practices, parental attitudes, parental knowledge, preventive dental care, saudi arabia, sugary snacks

## Abstract

Background: Preventive dental care is crucial for maintaining children's oral health and preventing dental diseases. Parental knowledge and practices significantly influence children's dental habits. However, gaps in awareness and inconsistent practices may contribute to the high prevalence of dental caries. In Saudi Arabia, the understanding of preventive dental care among parents remains underexplored.

Methods: A cross-sectional survey was conducted among 250 parents in Saudi Arabia using an online questionnaire. The survey assessed demographic characteristics, parental knowledge, practices, and attitudes toward preventive dental care for children. The data were analyzed using descriptive statistics.

Results: Most parents (43%) correctly identified the appropriate age for a child’s first dental visit. While 64% recommended dental check-ups every six months, 23% recommended yearly visits. A majority (68%) encouraged children to brush twice daily, though 16% reported that their children brushed only once. Regarding dietary habits, 19% of parents reported regular consumption of sugary snacks by their children. Time constraints (41%) and high costs (20%) were the most common barriers to regular dental visits. Over 85% of parents expressed interest in receiving more information on preventive dental care.

Conclusion: While many parents in Saudi Arabia recognize the importance of preventive dental care, there are significant gaps in both knowledge and practices, particularly regarding the frequency of dental visits and dietary habits. These findings highlight the need for targeted educational interventions to improve parental knowledge and practices, ultimately promoting better oral health outcomes for children.

## Introduction

Oral health is an essential component of overall well-being, and preventive dental care plays a critical role in maintaining good oral health throughout life. Dental caries (tooth decay) is one of the most prevalent chronic diseases globally, with children being particularly vulnerable to its development. The World Health Organization (WHO) has emphasized that dental caries is largely preventable through early interventions, including proper oral hygiene practices, diet modification, and regular dental visits. Despite these recommendations, studies have shown that many children suffer from preventable dental problems due to insufficient awareness and inconsistent preventive care practices among parents and caregivers [1,2].

Parental knowledge and attitudes are fundamental to the promotion of good oral health behaviors in children. Parents serve as the primary influence on their children's dental habits, including the frequency of brushing, the use of fluoride toothpaste, dietary habits, and the timely initiation of dental visits [3,4]. However, research indicates that many parents lack adequate knowledge regarding preventive dental care and often struggle to adopt practices that align with professional guidelines. Misconceptions about the appropriate age to begin dental visits, the frequency of check-ups, and the role of fluoride in preventing dental decay are common, potentially leading to delayed or inadequate preventive care [3,4].

In Saudi Arabia, a country with a growing population and evolving healthcare infrastructure, the prevalence of dental caries among children remains a significant public health challenge. Previous studies have reported a high incidence of dental caries in the primary dentition of Saudi children, with factors such as poor dietary habits, limited access to dental care, and a lack of awareness about preventive measures contributing to this burden [1-3]. Despite the availability of preventive dental services, a gap persists in the public’s understanding and practice of oral health guidelines. Given the importance of early intervention and prevention in addressing oral health issues, understanding the knowledge, attitudes, and practices of parents regarding their children's oral health is crucial for designing effective public health strategies [1,5].

Educational campaigns targeting parents are critical to improving dental health outcomes for children. Previous research has demonstrated that interventions aimed at increasing parental knowledge about preventive dental care can lead to improved oral hygiene practices and increased utilization of dental services [5-10]. However, there is limited data on parental knowledge and practices regarding preventive dental care in Saudi Arabia, particularly in urban and rural communities. This study seeks to fill this gap by assessing the current state of parental knowledge, practices, and attitudes toward preventive dental care for children in Saudi Arabia. The findings are expected to provide insights into the factors influencing dental health behaviors and inform future educational interventions aimed at improving children's oral health outcomes.

## Materials and methods

Study design

This was a cross-sectional survey-based study designed to assess parental knowledge and practices regarding preventive dental care for children. The study was conducted using an online questionnaire distributed through Google Forms (Mountain View, CA: Google LLC). The survey aimed to gather insights into the demographic characteristics of parents, their knowledge about preventive dental care practices, their own practices related to children's dental health, and their attitudes toward dental care for children.

Participants

The study was conducted among parents in Saudi Arabia. Participants were eligible if they were primary caregivers of children aged 0-18 years and had access to the Internet to complete the online survey. The target sample size was set at a minimum of 200 participants to ensure statistical power. A total of 250 participants completed the survey, providing a sufficient sample for analysis. Inclusion criteria for the study were parents or guardians with at least one child under 18 years of age, and participants had to provide informed consent before participating in the study. No incentives were provided for participation.

Survey instrument

The survey consisted of a self-administered questionnaire developed specifically for this study. It was designed to assess parental knowledge regarding preventive dental care, their practices in promoting dental health for their children, and their attitudes toward preventive care measures. The questionnaire included both multiple-choice questions (MCQs) and Likert scale questions. It was divided into four main sections. The first section gathered demographic information such as age, gender, educational background, number of children, and the age ranges of the children. The second section assessed participants’ knowledge of preventive dental care, including questions on the appropriate age to begin dental visits, the frequency of check-ups, fluoride toothpaste use, and factors contributing to cavities. The third section evaluated parents' practices regarding preventive care, including the frequency of encouraging their children to brush their teeth, assisting with brushing, the use of fluoride toothpaste, and their children’s consumption of sugary snacks and drinks. The final section examined the attitudes and barriers to preventive care, asking participants about their primary sources of information on dental care, perceived barriers to regular dental visits, and their overall interest in receiving more information on preventive dental care. The questionnaire was pre-tested for clarity and validity with a small group of parents before being distributed to the larger sample. Based on participant feedback, adjustments were made to improve the clarity of certain questions.

Data collection

The survey was distributed via a link to Google Forms, which was shared through various social media platforms, parent groups, and word-of-mouth. Participation was voluntary, and the survey was anonymous to ensure confidentiality. Participants were informed about the purpose of the study, the voluntary nature of their participation, and their right to withdraw at any point. The survey remained open for four weeks, during which participants were encouraged to complete the survey at their convenience.

Data analysis

Data collected from the online survey were exported into Microsoft Excel (Redmond, WA: Microsoft) for preliminary cleaning. Statistical analysis was performed using SPSS version 25 (Armonk, NY: IBM Corp). Descriptive statistics, including frequencies and percentages, were used to summarize demographic characteristics, knowledge, practices, and attitudes related to preventive dental care.

## Results

A total of 250 participants completed the survey, with 39% (97) identifying as male and 61% (153) as female. The majority of participants (36%, 91) were between 21 and 30 years of age, followed by those in the 31-40 years age group (29%, 72). A smaller proportion were in the 41-50 years (20%, 50), 51-60 years (10%, 25), and over 60 years (5%, 12) age groups.

Regarding educational background, 29% (73) of participants had completed high school, while 51% (128) held a bachelor’s degree. A further 17% (42) had obtained a master’s degree or higher, and 3% (7) selected "other" as their highest level of education. Most participants reported having two to three children (54%, 135), with 38% (95) having one child, and 8% (20) reporting four or more children. The majority of participants’ children were in the 0-3 years age group (55%, 138), followed by those in the four to six years age group (61%, 152), and 43% (108) had children aged seven to 12 years (Table 1).

**Table 1:** Demographic information of the study participants.

Demographic question	Response options	Frequency (%)
Gender	Male	97 (39%)
	Female	153 (61%)
Age group	21-30 years	91 (36%)
	31-40 years	72 (29%)
	41-50 years	50 (20%)
	51-60 years	25 (10%)
	Over 60 years	12 (5%)
Highest level of education	High school	73 (29%)
	Bachelor’s degree	128 (51%)
	Master’s degree or higher	42 (17%)
	Other	7 (3%)
Number of children	1 child	95 (38%)
	2-3 children	135 (54%)
	4 or more children	20 (8%)
Age range of children	0-3 years	138 (55%)
	4-6 years	152 (61%)
	7-12 years	108 (43%)
	13-18 years	80 (32%)

Knowledge of preventive dental care

When asked about the appropriate age to begin visiting a dentist, 43% (108) of participants indicated that visits should start at the age of one to two years, while 24% (59) selected three to four years of age as the preferred starting point. Only 16% (41) suggested five to six years, and 17% (42) were unsure. In terms of the frequency of dental check-ups, the majority of participants (64%, 160) recommended visits every six months, while 13% (33) recommended yearly visits. A smaller proportion (13%, 32) suggested visits every two years, and 10% (25) were unsure.

Regarding fluoride toothpaste, 52% (131) of participants recommended a rice-sized amount for children, 28% (71) suggested a pea-sized amount, and 12% (30) advised using a full brush-length amount. Interestingly, 7% (18) did not know the appropriate amount. In relation to factors contributing to cavities, 90% (226) of participants identified sugary snacks and drinks as a major factor, 70% (174) pointed to poor brushing habits, and 48% (121) believed that lack of dental visits contributed to cavities. Only 9% (23) of participants were unsure about the causes.

Participants were also asked about the appropriate age to start using dental floss. Forty-six percent (115) of respondents believed that it should be introduced when teeth begin touching, 22% (56) selected four to five years of age as the preferred starting point, and 18% (45) indicated when the child can brush alone. Fourteen percent (34) were unsure (Table 2).

**Table 2:** Knowledge of preventive dental care.

Question	Response options	Frequency (%)
Age to start visiting a dentist	1-2 years	108 (43%)
	3-4 years	59 (24%)
	5-6 years	41 (16%)
	I don’t know	42 (17%)
Frequency of dental check-ups	Every 6 months	160 (64%)
	Every 12 months	33 (13%)
	Every 2 years	32 (13%)
	I don’t know	25 (10%)
Recommended amount of fluoride toothpaste	Rice-sized amount	131 (52%)
	Pea-sized amount	71 (28%)
	Full brush-length amount	30 (12%)
	I don’t know	18 (7%)
Factors contributing to cavities	Sugary snacks and drinks	226 (90%)
	Poor brushing habits	174 (70%)
	Lack of dental visits	121 (48%)
	I don’t know	23 (9%)
When to start using dental floss	As soon as teeth are touching	115 (46%)
	4-5 years old	56 (22%)
	When they can brush alone	45 (18%)
	I don’t know	34 (14%)

Practices regarding preventive dental care

When asked about the frequency of encouraging children to brush their teeth, 68% (169) of participants indicated that they encouraged their child to brush twice a day, while 24% (60) encouraged brushing once a day. Only 4% (11) encouraged less than once a day, and another 4% (10) reported never encouraging brushing.

In terms of assistance with brushing, 47% (118) of parents reported always helping their children, 32% (81) helped sometimes, and 16% (41) stated that their children brushed on their own. A small proportion, 4% (10), did not know. The majority (75%, 187) of participants reported using fluoride toothpaste for their children, while 13% (33) did not, and 12% (30) were unsure.

Regarding sugary snacks and drinks, 19% (48) of participants reported that their children consumed them regularly, 57% (142) occasionally, and 22% (55) rarely. Only 2% (5) of participants stated that their children never consumed sugary snacks or drinks. When asked about the use of dental sealants or fluoride treatments, 17% (42) of participants reported using sealants, 15% (38) used fluoride treatments, and 65% (162) did not use either. Additionally, 3% (8) were unsure (Table 3).

**Table 3:** Practices regarding preventive dental care.

Question	Response options	Frequency (%)
Frequency of encouraging brushing teeth	Twice a day	169 (68%)
	Once a day	60 (24%)
	Less than once a day	11 (4%)
	Never	10 (4%)
Assist with brushing	Yes, always	118 (47%)
	Yes, sometimes	81 (32%)
	No, they do it themselves	41 (16%)
	I don’t know	10 (4%)
Use of fluoride toothpaste	Yes	187 (75%)
	No	33 (13%)
	I don’t know	30 (12%)
Consumption of sugary snacks/drinks	Regularly	48 (19%)
	Occasionally	142 (57%)
	Rarely	55 (22%)
	Never	5 (2%)
Use of sealants/fluoride treatments	Sealants	42 (17%)
	Fluoride treatments	38 (15%)
	Neither	162 (65%)
	I don’t know	8 (3%)

Attitudes and barriers to preventive dental care

Participants were asked about their primary source of information regarding dental care. Forty-two percent (104) cited dentists or hygienists as their primary source, while 21% (52) relied on the Internet or social media, and 18% (44) consulted family and friends. Other sources included pediatricians or healthcare providers (12%, 30) and books or pamphlets (5%, 13). Interestingly, 3% (7) of participants reported having no specific source of information.

Barriers to regular dental visits were also explored. The most common barrier was lack of time, with 41% (102) of participants identifying this as an issue. High cost was a barrier for 20% (49) of participants, while 24% (61) cited their child's fear of the dentist. Twelve percent (31) of respondents did not perceive any barriers to regular dental visits.

Regarding the importance of preventive dental care, 65% (162) of participants considered it very important, 28% (69) thought it was somewhat important, and 8% (19) did not consider it important. Finally, when asked about interest in receiving more information on preventive dental care, 85% (213) of participants expressed interest, 7% (18) were not interested, and 8% (19) were uncertain (Table 4).

**Table 4:** Attitudes and barriers to preventive dental care.

Question	Response options	Frequency (%)
Primary source of information	Dentist or hygienist	104 (42%)
	Internet or social media	52 (21%)
	Family and friends	44 (18%)
	Pediatrician or healthcare provider	30 (12%)
	Books or pamphlets	13 (5%)
	None or I don’t have a source	7 (3%)
Barriers to regular dental visits	High cost	49 (20%)
	Lack of time	102 (41%)
	Child’s fear of the dentist	61 (24%)
	None of the above	31 (12%)
Belief in importance of preventive dental care	Very important	162 (65%)
	Somewhat important	69 (28%)
	Not important	19 (8%)
Interest in more information	Yes	213 (85%)
	No	18 (7%)
	Maybe	19 (8%)

## Discussion

This study aimed to assess parental knowledge, practices, and attitudes regarding preventive dental care for children in Saudi Arabia. The findings revealed that while a majority of parents possessed a reasonable level of knowledge about preventive dental care, there were notable gaps in both practices and attitudes that could potentially influence children's oral health outcomes.

Our results indicate that most parents understood the importance of early dental visits, with 43% (108) correctly identifying the appropriate age to begin seeing a dentist (one to two years). This is consistent with current recommendations from dental associations, which advocate for the first dental visit by the age of one to ensure the early identification of oral health issues and the establishment of good oral hygiene practices [7,10]. However, a significant portion of parents (24%, 59) incorrectly believed that visits should begin later, at three to four years, while 17% (42) were unsure about the appropriate age. This finding underscores the need for more targeted educational campaigns to inform parents about the importance of early dental care.

In terms of dental check-up frequency, 64% (160) of parents recommended visits every six months, aligning with established guidelines for maintaining optimal oral health. However, a substantial proportion (23%, 57) either recommended yearly or less frequent visits, which may reflect a gap in understanding the recommended frequency of dental check-ups or the perceived lack of urgency in preventive care. This suggests an opportunity for increased public health education on the importance of regular dental visits to prevent the onset of dental caries and other oral diseases.

The study found that the majority of parents (68%, 169) encouraged their children to brush twice daily, a crucial habit for preventing dental diseases. Encouragement of proper brushing frequency is consistent with international recommendations, which stress the importance of brushing twice a day with fluoride toothpaste to prevent cavities. Interestingly, however, 4% (11) of participants reported encouraging brushing less frequently, and 4% (10) did not encourage brushing at all. This minority is concerning, as inconsistent oral hygiene habits can lead to the development of cavities and gum disease in children.

Regarding assistance with brushing, nearly half of the parents (47%, 118) reported always helping their children brush, which is in line with guidelines recommending parental supervision of young children’s brushing until they are capable of performing the task effectively on their own. However, 32% (81) of parents only sometimes assisted, and 16% (41) reported that their children brushed alone, which could reflect insufficient knowledge or time constraints among parents. This highlights the need for further efforts to emphasize the importance of parental involvement in young children's oral hygiene routines.

Another concerning finding was the high prevalence of sugary snack consumption, with 19% (48) of parents reporting that their children consumed sugary snacks and drinks regularly. This is a well-documented risk factor for the development of dental caries in children, and the high consumption of sugar-based foods and beverages is a growing public health concern globally. Strategies to reduce sugar intake, such as education on healthier dietary choices and the importance of limiting sugary snacks, should be incorporated into public health initiatives targeting parents [7-11].

The study also examined barriers to regular dental visits and found that lack of time (41%, 102) was the most commonly cited barrier, followed by high costs (20%, 49) and fear of the dentist (24%, 61). These findings are consistent with previous studies that have identified time and financial constraints as significant barriers to accessing dental care [11-13]. The perception of dental visits being expensive, coupled with a busy lifestyle, often results in delayed or infrequent visits, which can lead to untreated dental issues that may worsen over time. Public health initiatives addressing these barriers, such as promoting affordable dental care options and raising awareness about the long-term benefits of early dental visits, may help mitigate these obstacles.

In terms of attitudes, the majority of parents (65%, 162) considered preventive dental care to be very important. This is promising, as it indicates a strong belief in the value of maintaining children’s oral health. However, a notable proportion (28%, 69) believed it was only somewhat important, and 8% (19) did not consider it important. These findings suggest that while most parents recognize the importance of oral health, there remains a need for targeted education to reinforce the critical role of preventive care in promoting long-term oral health [13,14].

A significant number of parents (42%, 104) reported that their primary source of information on dental care was dentists or hygienists, which underscores the importance of dental professionals in educating families. However, 21% (52) relied on the internet or social media, and 18% (44) consulted family and friends for guidance. These non-professional sources may lead to the dissemination of misinformation or incomplete knowledge, highlighting the importance of strengthening the role of dental professionals in delivering accurate and evidence-based information. Moreover, 85% (213) of parents expressed interest in receiving more information on preventive dental care, emphasizing the opportunity for dental care providers to engage more proactively in educational outreach programs.

Limitations

This study has several limitations that should be considered when interpreting the results. First, the data were self-reported, which may lead to response bias, particularly for sensitive questions related to behaviors such as sugary snack consumption or dental care practices. Second, the study relied on an online survey, which may exclude parents who do not have internet access or are unfamiliar with digital platforms. Additionally, as a cross-sectional study, the data only provide a snapshot of parental knowledge and practices at one point in time, and causality cannot be inferred. Longitudinal studies are needed to assess changes in knowledge and practices over time.

## Conclusions

In conclusion, this study highlights significant gaps in parental knowledge and practices regarding preventive dental care for children in Saudi Arabia, despite a general awareness of its importance. While many parents recognize the need for early dental visits and encourage regular brushing, there are notable inconsistencies in practices such as frequency of dental check-ups and the management of sugary food intake. The findings suggest a critical need for targeted educational programs to improve parental understanding and adoption of best practices for children's oral health. By addressing these gaps through community outreach, school-based initiatives, and accessible dental education, we can promote better oral health outcomes for children and reduce the burden of preventable dental diseases in Saudi Arabia.
